# Rolling on the Chip: SARS-CoV-2 Detection by DNA Motors

**DOI:** 10.1021/acscentsci.4c00940

**Published:** 2024-07-12

**Authors:** Longjiang Ding, Na Liu

**Affiliations:** † 2. Physics Institute, University of Stuttgart, Pfaffenwaldring 57, 70569 Stuttgart, Germany; ‡ Max Planck Institute for Solid State Research, Heisenbergstraße 1, 70569, Stuttgart, Germany

COVID-19, a disease caused by the SARS-CoV-2 virus from the coronavirus family, has emerged as a global threat with profound implications for human health. The rapid and accurate detection of SARS-CoV-2 is paramount for promptly identifying infections and administering timely treatments, thereby controlling the spread of the virus.^1−3^ The premier diagnostic test for SARS-CoV-2, reverse transcription-quantitative PCR (RT-qPCR), can detect viral RNA at levels of as low as ∼10^2^–10^3^ copies/mL. However, this highly sensitive test typically requires a turnaround time of 10–15 h and is often conducted at centralized facilities, which can limit its accessibility and the speed of results.^4,5^ In contrast, antigen tests, such as the lateral flow assay (LFA), offer simplicity in operation with a readout time of just minutes. The LFA tests can be easily administered at the point of care without the need for specialized equipment or highly trained personnel. However, LFAs exhibit lower sensitivity, detecting viral loads ranging from 10^4^ to 10^6^ copies/mL, which can lead to a higher rate of false negatives than for RT-qPCR. Despite this limitation, their rapid turnaround and ease of use make them valuable for mass screening and early detection in various settings.^6^ Given the strengths and limitations of both RT-qPCR and antigen tests, the exploration and development of novel testing tools that complement existing methodologies are of great importance.

**Figure 1:**
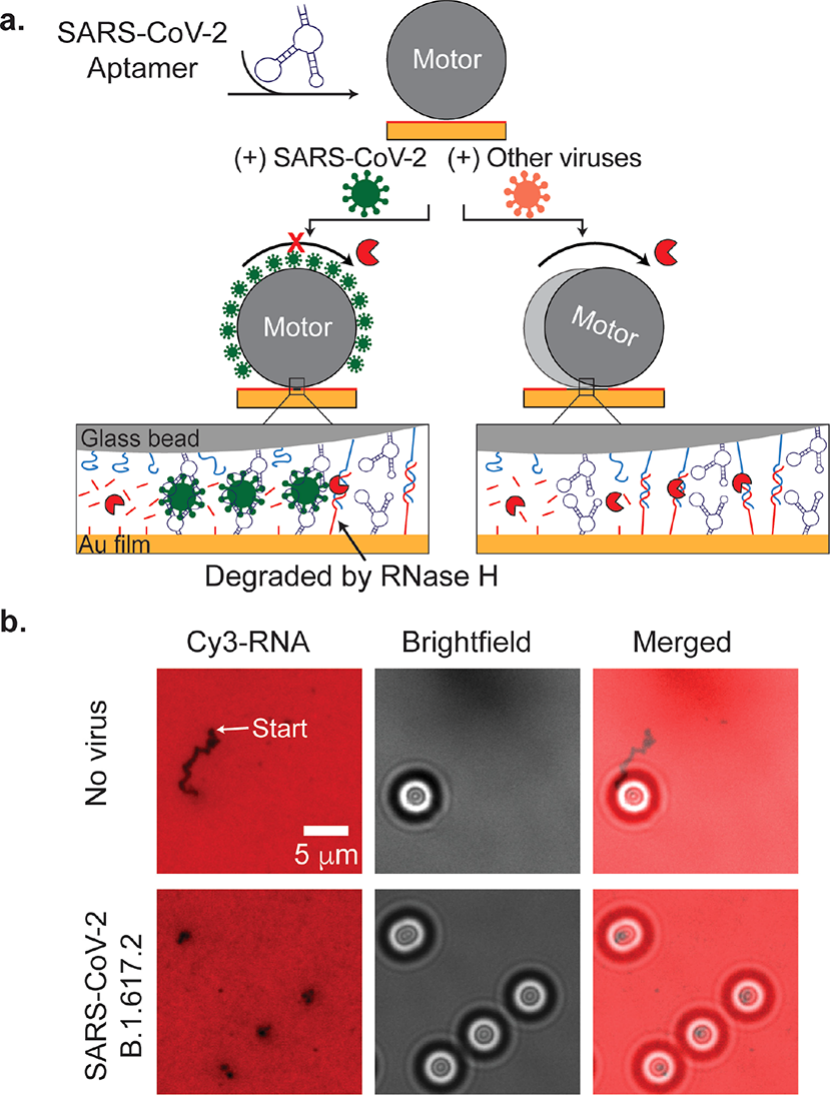
(a) Working mechanism of SARS-CoV-2 sensing by Rolosense. (b) Representative fluorescence and brightfield images of DNA motors for the detection of SARS-CoV-2 B.1.617.2 in artificial saliva. Reproduced with permission from ref (1). Copyright 2024. Published by the American Chemical Society.

In this issue of *ACS Central Science*, Salaita et al. report an interesting sensing platform, Rolosense, which leverages the rolling motion of DNA motors on a chip to detect SARS-CoV-2.^1^ The motor is a DNA-coated spherical particle (5 μm) that is hybridized to a chip surface modified with complementary RNA. By introducing ribonuclease H (RNaseH), the RNA/DNA duplexes can be specifically cleaved. The released chemical energy is converted to mechanical work, driving the motors to roll on the chip at speeds exceeding 1 μm/min.^7^ To sense SARS-CoV-2, both the DNA motors and the RNA chip were functionalized with SARS-CoV-2 virus-binding aptamers, which have a high affinity for the S1 subunit of spike protein, abundantly displayed on each virion (Figure 1a). When the viruses were bound to the motor and the chip surface, the force (∼100 pN)^8^ generated by the motor was insufficient to overcome the mechanical stability of the aptamer-target complex, causing the motor to halt (Figure 1b). Therefore, by monitoring the motion of the DNA motors, the presence of SARS-CoV-2 could be identified.

The authors showcased that Rolosense exhibited high specificity for SARS-CoV-2 among various respiratory viruses, including influenza A virus, as well as the seasonal common cold viruses HCoV OC43 and 229E. In particular, its sensitivity to SARS-CoV-2 B.1.617.2, WA-1, and the BA.1 variant in artificial saliva and exhaled breath condensate was at the level of 10^3^ copies/mL, which is clinically relevant in the early stages of infection. In terms of the limit of detection (LoD), Rolosense outperformed LFA-based tests, such as the BinaxNOW COVID-19 Ag Card (Abbott Diagnostics Scarborough, Inc.), which has an LoD of 10^5^ copies/mL for the BA.1 variant.^9^ Although Rolosense exhibited a weaker LoD compared to RT-qPCR, it offered the advantage of quantifying intact viral particles. This feature is significant as it avoids the detection of noninfectious or residual viral genetic materials. Moreover, virus detection using Rolosense could be performed with a smartphone in conjunction with a simple microscope setup, achieving an LoD of 10^3^ copies/mL.

Rolosense represents a valuable addition to the biosensor design toolkit, introducing a unique mechanical transduction mechanism that converts viral binding to the motion output of DNA motors. This approach opens up new possibilities for sensitive and specific viral detection. Engineering efforts aimed at optimizing the DNA and RNA densities on the motors and chip could further enhance the sensitivity and overall performance of the system. However, challenges remain, particularly the contamination and temperature sensitivity of RNaseH, which could lead to false-negative results. Addressing these issues or developing alternative methods to power the motion of DNA motors is crucial to improving reliability. Moreover, enabling direct monitoring of the motion using a smartphone, without the need for a dedicated microscope, would greatly enhance the practicality and accessibility of Rolosense in real-world applications. This would simplify the detection process, making it more user-friendly and portable. Future studies may focus on refining the system to achieve a balance among ease of use, compact design, cost-effectiveness, and rapid readout while preserving the high sensitivity and specificity that make Rolosense a promising diagnostic tool. Additionally, expanding the capabilities of Rolosense to detect a broader range of pathogens by using specific DNA aptamers to target different viruses could significantly increase its utility in various diagnostic contexts. Research into robust, field-deployable versions of Rolosense that can withstand varying environmental conditions will be essential for its application in diverse settings, from clinical laboratories to remote or resource-limited areas.
